# Phosphodiesterase‐5 Inhibition and Alzheimer's Disease Risk: A Mendelian Randomisation Study

**DOI:** 10.1111/acel.70265

**Published:** 2025-10-13

**Authors:** Marta Alcalde‐Herraiz, Benjamin Woolf, Junqing Xie, Emma Anderson, Dipender Gill, Ioanna Tzoulaki, Laura M. Winchester, James Yarmolinsky, Daniel Prieto‐Alhambra, Danielle Newby

**Affiliations:** ^1^ Centre for Statistics in Medicine and NIHR Biomedical Research Centre Oxford, NDORMS University of Oxford Oxford UK; ^2^ School of Psychological Science University of Bristol Bristol UK; ^3^ MRC Integrative Epidemiology Unit University of Bristol Bristol UK; ^4^ MRC Biostatistics Unit University of Cambridge Cambridge UK; ^5^ Division of Psychiatry University College of London London UK; ^6^ Department of Epidemiology and Biostatistics, School of Public Health Imperial College London London UK; ^7^ MRC‐PHE Centre for Environment, School of Public Health Imperial College London London UK; ^8^ Department of Hygiene and Epidemiology University of Ioannina Medical School Ioannina Greece; ^9^ Department of Psychiatry Warneford Hospital Oxford UK; ^10^ Department of Medical Informatics Erasmus University Medical Centre Rotterdam the Netherlands

**Keywords:** Alzheimer's disease, dementia, Mendelian randomisation, phosphodiesterase‐5, sildenafil

## Abstract

While preclinical studies suggest that Phosphodiesterase 5 (PDE5) inhibition may reduce cognitive impairment, findings from observational studies on whether PDE5 inhibitors reduce Alzheimer's disease (AD) risk have been inconsistent. We performed a two‐sample *cis*‐Mendelian Randomisation (MR) analysis to estimate the causal effect of PDE5 inhibition on AD risk. The analysis was performed across four different genome‐wide association studies (GWAS) of AD to enhance reliability through triangulation. Additionally, a sex‐stratified MR analysis using data from UK Biobank was performed to assess potential sex‐specific effects. No evidence of a causal association between PDE5 inhibition and AD risk was found in the main analyses. Similar findings were obtained in the sex‐stratified analysis. Our study uses genetic data to triangulate the evidence and suggests that PDE5 inhibitors are unlikely to decrease the risk of AD. Further research is needed to thoroughly understand the impact of PDE5 inhibitors on the risk of Alzheimer's disease.

## Graphical abstract

Preclinical studies suggest PDE5 inhibitors may improve cognition, but observational evidence on Alzheimer's disease (AD) risk is mixed. We performed a *cis*‐Mendelian randomisation analysis across four AD genome‐wide association studies (GWAS) datasets and a sex‐stratified analysis using UK Biobank data. No genetic evidence was found that PDE5 inhibition reduces AD risk, suggesting PDE5 inhibitors are unlikely to reduce the risk of AD, and highlighting the need for further research.

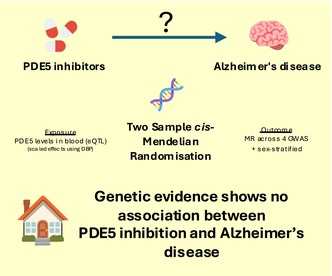

## 1 Introduction

Alzheimer's disease (AD) is a progressive neurodegenerative disorder and the most common cause of dementia. It is characterised by the accumulation of beta‐amyloid plaques and neurofibrillary tangles in the brain, leading to a gradual deterioration of cognitive function and memory (NHS 2024). With the global increase in life expectancy, AD is rapidly emerging as a significant public health threat worldwide (Alzheimer's's 2016).

Recently, the Medicines and Healthcare products Regulatory Agency (MHRA) in the UK approved Lecanemab for treating adults in the early stages of AD (GOV.UK 2024). While the treatment has been shown effective for slowing the disease progression, the National Institute for Health and Care Excellence (NICE) has not recommended its availability on the NHS, arising concerns about its cost‐effectiveness (NICE 2024). There is therefore still a scarcity of drugs that can effectively treat or prevent AD.

Several studies have identified numerous modifiable risk factors that may be associated with AD (Livingston et al. 2024), offering the potential opportunity for intervention through drug repurposing strategies (Pushpakom et al. 2019). This strategy aims to identify new therapeutic uses for existing drugs that have already been approved. In terms of cost‐effectiveness, reduced drug‐development time, and lower risk of failure, repurposing presents a highly advantageous strategy. Within this context, antihypertensive and related medications have been previously highlighted as promising candidates for AD prevention (Hughes et al. 2020). Specifically, phosphodiesterase 5 (PDE5) inhibitors have gained growing interest due to their potential neuroprotective effects (Webb et al. 2024).

PDE5 inhibitors (i.e., sildenafil, vardenafil, tadalafil, and avanafil) are mainly used for the treatment of erectile dysfunction (ED) and pulmonary arterial hypertension (PAH) (Barnett and Machado 2006; Levine 2004). PDE5 is an enzyme present in smooth muscle cells whose inhibition has been shown to induce vascular smooth relaxation and vasodilatation. Simultaneously, this results in a reduction of diastolic blood pressure (Kloner 2004). Some preclinical models have also highlighted the potential of PDE5 inhibitors on improving memory function (Zuccarello et al. 2020), although these results were not observed in subsequent clinical studies.

Conflicting results have also been reported in observational studies (Desai et al. 2022; Fang et al. 2021), where confounding, time‐related bias or reverse causation can play a key role. Alternatively, Mendelian randomisation (MR) can offer a robust approach to investigate causal relationships, overcoming some of the limitations of observational research, as well as allowing for triangulation of evidence (Schmidt et al. 2020; Smith and Ebrahim 2003). Specifically, drug target MR is an analytical method that uses genetic variants within or near the gene encoding the risk factor (*cis*‐variants) as instruments to proxy the exposure (Gill et al. 2021; Schmidt et al. 2020). Since alleles are randomly allocated during meiosis, this approach can minimise the risk of confounding that typically affects traditional observational studies, provided that all underlying MR assumptions are met.

In this study, we performed a two‐sample *cis‐*MR analysis to estimate the causal effect of genetically proxied PDE5 inhibition and the risk of AD. To investigate this, we used diastolic blood pressure as a surrogate biomarker. Furthermore, we also conducted a stratified‐by‐sex MR analysis, where we used UK Biobank (UKB) to identify AD cases.

## 2 Materials and Methods

### 2.1 Study Design

**FIGURE 1:**
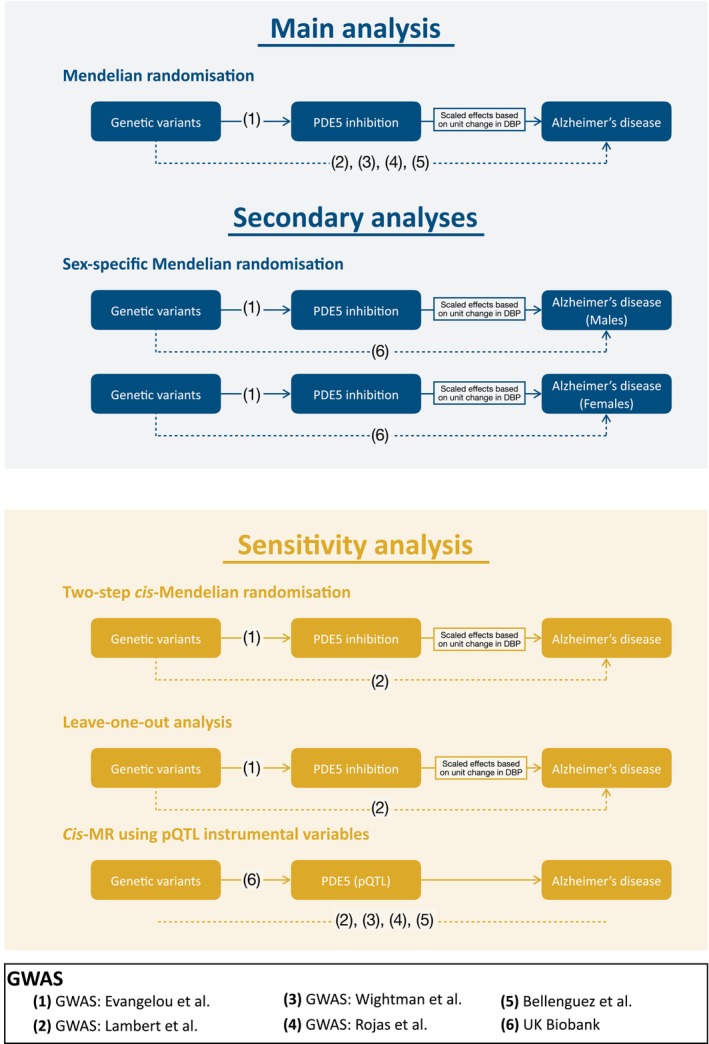
Scheme of the study design. GWAS, genome‐wide association study.

We performed a two‐step *cis*‐Mendelian Randomisation analysis to estimate the causal effect between genetically proxied PDE5 inhibition and AD risk. We scaled the effect of PDE5 inhibition based on a surrogate biomarker—diastolic blood pressure (Schmidt et al. 2020; Schmidt et al. 2022). We used four different genome‐wide association studies (GWAS) of AD, each one differing by the sample size, heritability value, and the use of by‐proxy cases. Additionally, we performed a sex‐stratified analysis using the UKB dataset to identify AD cases. Figure 1 summarises the study design.

### 2.2 Data Sources

#### 2.2.1 Variant‐Risk Factor Estimates

Variant‐exposure estimates were extracted from a GWAS of diastolic blood pressure (Evangelou et al. 2018). We used diastolic blood pressure (DBP) instead of systolic blood pressure (SBP) as PDE5 inhibition is known to have a greater impact on the former (Kloner 2004). Evangelou et al. GWAS meta‐analysed 77 cohorts (*n* = 757,601) participating in the International Consortium for Blood Pressure Genome (ICBP) (Wain et al. 2017) and UKB (Sudlow et al. 2015). Blood pressure for each individual in the participating cohorts was measured in mmHg using either manual or automated readings. All participating cohorts adjusted for age, age^2^, sex, body mass index, and study‐specific covariates. UKB cohort further corrected BP measures for those with self‐reported medication use. Further information about study design, participants, and genotype quality control of this GWAS can be found in the original publication.

GWAS estimates were measured in mmHg change per effect allele, and SNP positions were reported in hg19/GRCh37 coordinates.

#### 2.2.2 Variant‐Outcome Estimates

Genetic variant‐outcome associations were derived from the AD GWAS published by Lambert et al. (2013), which conducted a two‐stage meta‐analysis of GWAS in individuals of European ancestry. We used estimates from stage 1, which consisted of 54,162 participants (17,008 AD cases and 37,154 controls) containing 7,055,881 genotyped and imputed variants. Further details of the databases included in this meta‐analysis can be found in Note S1 and Table S1. As secondary outcomes, we used three different AD GWAS: de Rojas et al. (2021) (*N* = 409,435), Bellenguez et al. (2022) (*N* = 487,511) and Wightman et al. (2021) (*N* = 398,108). All of them included only European participants. More details about the study populations can be found in the respective original publications and in Note S1 and Table S1.

We included these additional AD GWAS as secondary outcomes to validate our main study results. We did this due to the following reasons: (1) although they have bigger sample sizes, these AD GWAS have smaller SNP‐based heritability values compared to Lambert AD GWAS; (2) the AD GWAS conducted by Rojas and Bellenguez contain a proportion of by‐proxy cases for AD and (3) Rojas and Bellenguez GWAS included UKB, as well as Evangelou blood pressure GWAS, and we wanted to avoid sample overlap as it can lead to biased results in the presence of weak instruments (Sadreev et al. 2021).

All GWAS estimates were reported on the log(OR) scale. SNP coordinates were in hg19/GRCh37 assembly, except Rojas et al. GWAS, which was in the hg38/GRCh38 assembly. We aligned the coordinates of this GWAS from hg38/GRCh38 to hg19/GRCh37 using LiftOver (Hinrichs et al. 2006).

In the sex‐specific analysis, we used UKB (Sudlow et al. 2015) to estimate the variant‐outcome effects. UKB is a large population‐based cohort study of over 500,000 participants aged 40–69 at recruitment (2006 and 2010). UKB collected lifestyle data and biological samples for genotyping (Bycroft et al. 2018). Genotyping, performed by Affymetrix, includes 784,256 autosomal variants. Imputation was done using the Haplotype Reference Consortium and UK10K panels, covering 93 million SNPs. Health outcomes, including dementia and AD, were ascertained using validated algorithms combining baseline data and linked hospital and death records, with follow‐up data until December 2022 (https://biobank.ndph.ox.ac.uk/showcase/refer.cgi?id=460). In total, there are around 10,000 dementia cases recorded in UKB.

### 2.3 Statistical Analysis

#### 2.3.1 Instrument Selection

Instrumental variables used in MR must (1) be associated with the exposure, (2) not be associated with the outcome through confounding pathways, and (3) not affect the outcome except via the exposure (Burgess et al. 2019). Instrumental variables to proxy PDE5 inhibition were obtained from a previous study that also targeted PDE5 inhibition (Woolf et al. 2023). This study selected five *cis*‐genetic variants within the PDE5 gene (chromosome 4, position in GRCh37/hg19 120,415,550–120,550,146). These variants were associated with PDE5 gene expression (expression quantitative trait loci, eQTL) in blood at genome‐wide significance (*p*‐value < 5 × 10^−8^) and clumped using the *p*‐values of their associations with diastolic blood pressure (*r* ^2^ < 0.35, window = 10,000 kilobases). Instruments from this study were validated using two positive control outcomes (erectile dysfunction and pulmonary hypertension).

These five variants extracted from Woolf et al. study were used as instruments for this study (Table 1). The variant with the strongest association (i.e., lowest *p*‐value) was rs66887589 (*β* = −0.16 respect allele T, 95% CI = −0.2 to −0.12). Variant rs17355550 was the one with the weakest association with DBP (*β* = −0.14 respect allele C, 95% CI = −0.23 to −0.04).

**TABLE 1:** Single nucleotide polymorphisms employed as instruments for the Mendelian randomisation analysis.

SNP	Effect allele	Other allele	EAF	Diastolic blood pressure (*N* = 757,601)					eQTL (*N* = 31,684)
				Beta (mmHg)	SE	*p*	Sample size	*F*‐statistic^a^	*p*
rs66887589	C	T	0.48	0.16	0.02	2e‐20	754,581	86	1.9e‐40
rs10050092	C	T	0.34	−0.13	0.02	9e‐13	754,583	51	1.5e‐18
rs12646525	T	C	0.22	−0.10	0.02	7e‐06	746,319	20	1.5e‐9
rs80223330	A	G	0.14	0.10	0.03	0.0001	749,960	15	2.1e‐34
rs17355550	C	T	0.03	−0.14	0.05	0.004	746,320	8	3.5e‐8

Abbreviations: EAF, effect allele frequency; eQTL, expression quantitative loci; SE, standard error; SNP, single nucleotide polymorphism.

^a^ *F*‐statistic was calculated using the Cragg‐Donald *F*‐statistic (Burgess, Thompson, and Crp Chd Genetics Collaboration 2011).

#### 2.3.2 Main Analysis

Mendelian randomisation estimates were calculated using the inverse‐variance weighted method accounting for correlation between variants (Burgess et al. 2017). We used the linkage disequilibrium matrix corresponding to European ancestry participants with the 1000 Genomes reference panel phase 3. Exposure alleles and outcome alleles were harmonised with respect to the linkage disequilibrium matrix. All MR estimates were reported as OR. We scaled our estimates to represent a daily 100 mg dose of sildenafil, knowing that this amount decreases 5.5 mmHg DBP.

#### 2.3.3 Sex‐Stratified Analysis

As AD is more prevalent in women compared to men (Andersen et al. 1999), we repeated the main analysis separately in females and males. We used the same variant‐risk factor GWAS as in the main analysis (described in section Methods/Data sources/Variant‐risk factor estimates), as previous research showed that SNP effects were identical for both the mixed and the specific sex analysis (Woolf et al. 2024). We used UKB to calculate genetic variants‐AD associations separately for both females and males.

We used the algorithmically defined dementia types to restrict our UK Biobank cohort to participants without other forms of dementia (frontotemporal, vascular, or all causes of dementia—including only those with AD). AD cases were classified as those with an AD report, whereas AD controls were those without one. All UKB variable field IDs used in the study can be found in Table S2.

We estimated the effect size of the genetic instruments on the algorithmically defined AD (separately for each sex) using a logistic regression. We used age at first assessment, the genetic batch, and the first 10 genetic principal components as covariates in the model. Beta coefficients obtained from the two different regressions were used later for computing the MR estimates.

MR effects were reported as OR per 5.5 mmHg decrease in DBP.

### 2.4 Sensitivity Analysis

#### 2.4.1 Two‐Step *Cis‐*Mendelian Randomisation

To account for any potential horizontal pleiotropic effects, we performed two‐step *cis*‐MR. This approach employs a two‐step mediation strategy to account for potential pleiotropic pathways by adjusting variant‐outcome associations (Woolf et al. 2022). Hence, this approach is used to adjust our MR estimates for any effect mediated by other related traits.

We adjusted our analysis for body mass index (BMI), as its inclusion as a covariate in the DBP GWAS can induce collider bias (Hartwig et al. 2021).

#### 2.4.2 Leave‐One‐Out Analysis

To assess whether the results from the main analysis were driven by a single instrument, we performed leave‐one‐out analysis (Davies et al. 2018). In this approach, one SNP from the instrumental set is removed and the estimated causal effect is re‐calculated. We performed this analysis for each one of the AD GWAS included in this study.

#### 2.4.3 *Cis‐* MR Using Protein Quantitative Loci (pQTL) Variants

To assess whether the results were consistent if instrumental variables were selected based on pQTL, we performed a *cis*‐MR analysis using two uncorrelated SNPs in PDE5 associated with circulating levels of PDE5 protein identified from UKB‐PPP (Tang et al. 2025).

### 2.5 Software and Implementation

We used R (version 3.2) for this study. The main packages used included *TwoSampleMR* (Hemani et al. 2018), *TwoStepCisMR* (Woolf et al. 2022), *coloc* (Giambartolomei et al. 2014), liftOver (Hinrichs et al. 2006), dplyr (Wickham et al. 2023), and ggplot2 (Wickham 2016). This manuscript was written according to the STROBE‐MR reporting guidelines (Skrivankova et al. 2021).

All the analytical code can be found in the public GitHub repository: https://github.com/oxford‐pharmacoepi/PDE5_AD_MendelianRandomisation.

## 3 Results

### 3.1 Main Analysis

**FIGURE 2:**
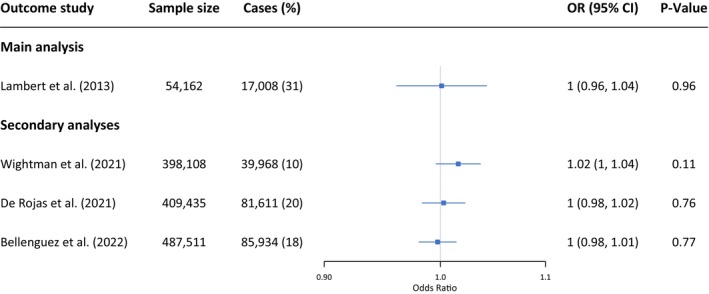
Results from the Mendelian randomisation. Odds ratio is scaled to represent a 100 mg dose of sildenafil (which reduces 5 mmHg diastolic blood pressure). CI, confidence interval; OR, odds ratio.

We found little evidence of an association between genetically proxied PDE5 inhibition and AD risk using Lambert et al.'s GWAS (OR = 1.00, 95% confidence interval 0.96–1.04, *p*‐value = 0.96). These results were consistent when using the other AD GWAS data (Figure 2).

Refer to Table S3 to see the linkage disequilibrium matrix employed. Harmonised variant‐risk factor and variant‐outcome effects can be found in Table S4. SNPs' effect on the exposure against SNPs' effects on the outcome (Lambert et al.) can be found in Figure S1.

### 3.2 Sex‐Stratified Analysis

From the 502,230 participants in UKB, we excluded 6153 participants because they were diagnosed with vascular dementia, frontotemporal dementia or other forms of dementia (Figure S2). After further restricting to individuals with genotyped data, we obtained a cohort of 262,037 females with 2030 AD cases, and a cohort of 220,352 males with 1722 cases. Baseline characteristics of the respective cohorts can be found in Table S5.

MR results for both sexes were consistent with the main analysis (Figure S3), showing no association between genetically proxied PDE5 inhibition and AD risk (Male: scaled OR = 1.04, 95% CI = 0.97 to 1.11, *p*‐value = 0.3; Female: scaled OR = 0.95, 95% CI = 0.88–1.03, *p*‐value = 0.2).

### 3.3 Sensitivity Analyses

#### 3.3.1 Two‐Step *Cis‐*Mendelian Randomisation

Results from the two‐step *cis‐*MR were consistent with the main findings (Table S6), suggesting that BMI did not induce bias in our results.

#### 3.3.2 Leave‐One‐Out

There was little evidence to suggest that any of the instruments driven to the obtained results was found in the leave‐one‐out analysis. All results were consistent with the main findings (Table S7).

#### 3.3.3 pQTL Variants

Results from the *cis*‐MR using pQTL instrumental variables were aligned with the main findings (Table S8).

## 4 Discussion

In this study, we used drug target MR to investigate the association between genetically proxied PDE5 inhibition and the risk of AD. Using DBP to scale PDE5 inhibition, our analysis showed little evidence of an effect of PDE5 inhibition on AD risk using a variety of AD GWAS datasets. Results stratifying by sex also showed no evidence of a causal association.

PDE5 is an enzyme responsible for degrading cyclic guanosine monophosphate (cGMP) (Tropea et al. 2022), a molecule that activates protein kinase G (PKG). PKG plays a key role in the regulation of smooth muscle relaxation, particularly in blood vessels. In addition to its vascular effects, the cGMP/PKG signalling has also been shown to play a role in neuronal plasticity (Bollen et al. 2014; Ota et al. 2008; Prickaerts et al. 2004). Animal models have shown the potential of sildenafil enhancing memory and cognitive function (Cuadrado‐Tejedor et al. 2011; Hosseini‐Sharifabad et al. 2012; Prickaerts et al. 2002; Puzzo et al. 2009) while having other neuroprotective effects, such as improving synaptic plasticity (Palmeri et al. 2013), or reducing amyloid burden (Puzzo et al. 2009). However, clinical studies' results regarding sildenafil efficacy to enhance cognition have not yet been conclusive (Zuccarello et al. 2020), although it has been recently shown to improve brain blood flow (Webb et al. 2024).

Interestingly, observational studies have also found contradictory results. Fang et al. (2021) developed an endophenotype network analysis where sildenafil was identified as a candidate drug in AD. They further conducted an observational study that suggested a reduction of 69% in the risk of AD among sildenafil users compared to nonusers. This result was not aligned with a new‐user active comparator cohort study conducted by Desai et al. (2022), where they did not find any evidence of an association between sildenafil and endothelin receptor antagonist users in people with PAH. Strengths, limitations and differences between the previous two observational studies have been extensively discussed elsewhere (Desai et al. 2022; Her et al. 2023; Newby 2022), mainly pointing to issues regarding the target population and the study design, which contributed to divergent associations in both studies.

Our study presents genetic evidence that supports Desai et al.'s results. However, there are significant differences in the interpretation of both studies. First, Desai and colleagues included other dementias (vascular dementia, senile, pre‐senile, or unspecified dementia) rather than AD to define the outcome, which can bias the results to the null if sildenafil has only impact on AD. In our study, we used different GWAS studies for AD, each one with a different definition. For example, the main study outcome (Lambert et al. 2013) did include (although in small proportion) participants with mixed AD and vascular dementia (Note S1) to define AD, as well as Bellenguez et al. (2022) definition. However, Wightman et al. (2021) and de Rojas et al. (2021) used a more specific definition for AD. Second, they restricted the study population to participants with PAH to minimise the risk of confounding by indication, significantly reducing the study population sample size. This can result in a lack of statistical power and generalisability to the general population, not only because of the sample size but also because, given the high mortality rate among medicated PAH patients, it can be that participants did not live long enough to develop AD. In our study, we used Mendelian randomisation, which assumes that alleles are randomised at meiosis; hence, we could study the impact of genetically proxied PDE5 inhibition, instrumented in a wider European population. Third, whereas they studied the impact of sildenafil usage on AD, our study did not assess that. Instead, we studied the effect of having lower levels of PDE5 over a lifelong time.

AD has been reported to be more prevalent in women (Andersen et al. 1999), whereas sildenafil is mainly prescribed to men. Hence, we performed a sex‐stratified analysis using data from UKB to estimate the variant‐outcome associations independently for males and females. Our findings indicated that genetically proxied PDE5 inhibition was not associated with a reduced risk of AD in either sex. However, both analyses were influenced by a significant case–control imbalance ratios, with only around 0.8% of participants being classified as cases. Hence, our sex‐stratified analysis can be underpowered to detect any association.

A previous observational study conducted only in men diagnosed with ED had identified an association between the use of PDE5 inhibitors and decreased risk of AD (Adesuyan et al. 2024). The cohort study compared users of PDE5 inhibitors with non‐users, based on the assumption that patients continued the treatment after the initial prescription. However, PDE5 inhibitors for ED are typically taken when required rather than regularly. This intermittent use challenges the assumption that participants consistently followed up with the treatment once prescribed, potentially impacting the study's conclusions.

Our study has several strengths that enhance the robustness of our findings. First, we used MR to triangulate evidence from previous observational evidence, which is known to be less susceptible to unobserved confounding and reverse causation, compared to traditional observational studies (Gill et al. 2021; Schmidt et al. 2020). Second, we used instrumental variables that have been previously tested and validated using positive control outcomes, enhancing their reliability. Third, we included different AD definitions, each one prone to different biases, in the main analysis and obtained consistent evidence, ensuring the robustness of our results. Additionally, we further performed a two‐step *cis‐*MR to adjust for potential pleiotropic effects, where results were also consistent with the main analysis.

There are some limitations that must be considered when interpreting our results. First, by using MR, we are studying the effect of small lifelong effects of lower PDE5 levels on AD. However, PDE5 inhibitors are typically administered for a shorter time, in higher doses and at a specific point in time. Thus, the effect estimates from this study should not be interpreted as the effect of the pharmacological intervention, but as an approximation of the direction of the causal effects. Second, we only included European populations in our study (although in the sex‐specific analysis we did not restrict to increase the sample size, the proportion of non‐white individuals was much smaller compared to white individuals). Therefore, our results are potentially not generalisable to other populations. Third, we acknowledge the interest in subgroup analyses (by age, ancestry, or clinical subtypes such as APOE‐ε4 carriers' status), which could indeed reveal subgroup‐specific effects. However, these analyses were not feasible in the present study. However, it could be performed when more data become available to ensure adequately powered analyses. Fourth, our model assumes that the effects of PDE5 inhibition are linear across the dose–response range. Fifth, although we included different AD GWAS with different sources of bias, most of them relied on clinical diagnostic rather than postmortem autopsy (which is known to be the gold standard to detect the underlying cause of dementia). Hence, heterogeneity in our outcome might influence the results, as different types of dementia can have different risk factors (Anderson et al. 2024).

Our study provides genetic evidence that PDE5 inhibitors are unlikely to decrease the risk of AD. Findings from this study have significant implications for our understanding of AD pathophysiology and the identification of PDE5 inhibitors' potential therapeutic targets.

## Author Contributions

M.A.‐H., D.N., B.W., D.G., I.T., and J.Y. designed the study. M.A.‐H. performed the data analysis and drafted the first version of the manuscript. B.W., J.X., E.A., D.G., I.T., L.M.W., J.Y., D.P.‐A., and D.N. reviewed critically the content of the manuscript, edited further versions and approved the final version and revisions for submission.

## Conflicts of Interest

D.G. is the Chief Executive Officer of Sequoia Genetics, a private limited company that works with investors, pharma, biotech, and academia by performing research that leverages genetic data to help inform drug discovery and development. D.G. has financial interests in several biotechnology companies. D.P.‐A.'s department has received grants from Amgen, Chiesi‐Taylor, Lilly, Janssen, Novartis, and UCB Biopharma. D.P.‐A.'s research group has received consultancy fees from AstraZeneca and UCB Biopharma. Amgen, Astellas, Janssen, Synapse Management Partners, and UCB Biopharma have funded or supported training programmes organised by D.P.‐A.'s department. All other authors declare no conflicts of interest.

## Supporting information


**Appendix S1:** acel70265‐sup‐0001‐AppendixS1.docx.

## Data Availability

All GWAS used are publicly available (see references to original publications and Table S1). UK Biobank individual‐level source data can be accessed by applying for access at http://ukbiobank.ac.uk/register‐apply/. This research has been conducted using the UK Biobank Resource under application number 98358.
